# FOXP3^+^ T Regulatory Cell Modifications in Inflammatory Bowel Disease Patients Treated with Anti-TNF**α** Agents

**DOI:** 10.1155/2013/286368

**Published:** 2013-08-26

**Authors:** Luisa Guidi, Carla Felice, Annabella Procoli, Giuseppina Bonanno, Enrica Martinelli, Manuela Marzo, Giammarco Mocci, Daniela Pugliese, Gianluca Andrisani, Silvio Danese, Italo De Vitis, Alfredo Papa, Alessandro Armuzzi, Sergio Rutella

**Affiliations:** ^1^Department of Internal Medicine, Inflammatory Bowel Disease Unit, Complesso Integrato Columbus, Catholic University, Largo Gemelli 8, 00168 Rome, Italy; ^2^Department of Gynaecology, Catholic University, Rome, Italy; ^3^Inflammatory Bowel Disease Unit, IRCCS Clinical Institute Humanitas, Rozzano, Milano, Italy; ^4^Department of Paediatric Haematology/Oncology, IRCCS Bambin Gesù Children's Hospital, Rome, Italy

## Abstract

Treg modulation has been hypothesized as one of the mechanisms by which antitumor necrosis factor **α** (TNF**α**) agents exert their action in rheumatoid arthritis (RA) and inflammatory bowel disease (IBD). However, data in IBD are still conflicting. We evaluated CD4^+^CD25^+^FOXP3^+^ (Tregs) by flow cytometry in peripheral blood from 32 adult IBD patient before (T0) and after the induction of anti-TNF**α** therapy (T1). Eight healthy controls (HCs) were included. We also evaluated the number of FOXP3^+^ cells in the lamina propria (LP) in biopsies taken in a subset of patients and controls. Treg frequencies were significantly increased in peripheral blood from our patients after anti-TNF**α** therapy compared to T0. T1 but not T0 levels were higher than HC. The increase was detectable only in clinical responders to the treatment. A negative correlation was found among delta Treg levels and the age of patients or disease duration and with the activity score of Crohn's disease (CD). No significant differences were found in LP FOXP3^+^ cells. Our data suggest the possibility that in IBD patients the treatment with anti-TNF**α** may affect Treg percentages and that Treg modifications may correlate with clinical response, but differently in early versus late disease.

## 1. Introduction

Inflammatory bowel diseases (IBDs) include Crohn's disease (CD) and ulcerative colitis (UC), which are chronic inflammatory illnesses of the intestinal tract. Their etiology is unknown, and the pathogenesis is not yet fully understood, but an immune dysregulation associated with loss of tolerance to the gut flora seems to be the main mechanism leading to uncontrolled inflammatory activation and subsequent tissue damage in genetically predisposed subjects. 

A variety of cells with regulatory properties have been described [1]. Among them, CD4^+^CD25^+^ T regulatory cells (Tregs) play a crucial role in the maintenance of self-tolerance and the prevention of autoimmune diseases. They constitute up to 6% of the CD4^+^ T cells in human peripheral blood of healthy adult volunteers [2] and also reside in the human intestinal lamina propria [3]. FOXP3 is a member of the *forkhead*-winged helix family of transcription factors expressed by CD4^+^CD25^+^ T cells and represents an important functional marker of their activity [4]. 

Since 1993, a role for a CD4 subset (CD45 RBlo) in the prevention of the colitis induced in immunodeficient mice by the transfer of naïve T cells has been demonstrated [5, 6]. This subset has been subsequently characterized as the CD4^+^CD25^+^FOXP3^+^ fraction, and it has been shown how the transfer of these cells does not only prevent but also cures experimental colitis in mice [7, 8]. The confirmation of FOXP3 function derives from a recent discover: a mutation of the *FOXP3* gene may lead to a pediatric syndrome characterized by the association of several different autoimmune diseases (IPEX Syndrome: immune dysfunction, poliendocrinopathy, enteropathy, and X-linked) [9]. 

In humans, the protective role of Tregs against autoimmune disorders has been largely studied evaluating their number and their activity in several diseases, such as type 1 diabetes [10], multiple sclerosis [11], systemic lupus erythematosus [12], rheumatoid arthrithis (RA) [13], psoriasis [14], and IBD. Recently, the encouraging results of a phase 1/2a clinical trial of antigen-specific regulatory T cell therapy (another subset of T regulatory cells, IL-10 producing Tr1) in patients with refractory Crohn's disease have been reported [15].

New concepts are recently emerging from Treg assessment during biological therapy with anti-TNF*α* (Tumor Necrosis Factor *α*) agents. TNF*α* is a cytokine that plays a crucial role in the development and maintenance of chronic inflammation in several immune-mediated disorders. Thus the use of anti-TNF*α* agents was demonstrated to be efficacious to achieve and keep clinical remission principally in IBD, rheumatoid arthritis, and psoriasis [16–18]. However, there is still a considerable percentage of patients who primarily do not respond or lose benefit from these treatments over variable time. 

The analysis of Tregs in these particular settings is aimed at evaluating their possible function as marker of response to treatment as well as considering their role in the pathogenesis of these diseases and finally hypothesizing new therapeutic strategies that could involve the Treg pathway. Studies in rheumatoid arthritis (RA) have shown that infliximab (a chimeric anti-TNF*α*) and methotrexate may lead to a significant rise in the number of peripheral blood Tregs in patients responding to this therapy [19] and induce *in vitro* the differentiation of a population of Tregs expressing FOXP3 through conversion of CD4^+^CD25^−^ T cells [20]. 

In IBD patients, data on Tregs have been somewhat conflicting. Recent studies showed that anti-TNF*α* treatment increases Treg level in the peripheral blood of IBD patients [21–23], especially in clinical responders [22, 23]. However, this has not been confirmed by the study of Grundström et al. [24]. At variance, Veltkamp described reduced frequencies of peripheral blood Tregs in active IBD [25].

Data concerning the amount of FOXP3^+^ T cells in the inflamed gut are also still unclear, with more reports of an increase in the number of these cells [21, 25–28] but also unchanged [24] or decreased [29] counts of mucosal Tregs. The main objective of our study was to assess the frequency of FOXP3^+^CD4^+^CD25^+^ T regulatory cells in peripheral blood and of FOXP3^+^ cells in mucosal biopsy specimens from IBD patients before and after different anti-TNF*α* therapies, correlating the results with clinical response, C-reactive protein (CRP) levels, and age and duration of disease. 

## 2. Materials and Methods

### 2.1. Patients

Thirty-two consecutive IBD adult patients with a clinical indication for anti-TNF*α* treatment were studied in 2007 and 2008. Twenty-five patients were affected by active Crohn's disease and seven by active ulcerative colitis. Sixteen healthy controls were also studied for comparison, analyzing peripheral blood in eight subjects and histological samples in the other eight. The protocol was approved by the local Ethical Committee. Fifteen CD and the seven UC patients were treated with infliximab (a chimeric anti-TNF*α* monoclonal antibody) with 5 mg/kg intravenous infusions at week 0, 2, 6, and then every 8 weeks; six CD patients received certolizumab pegol (a human pegilated anti-TNF*α* Fab') with 400 mg subcutaneous injections at week 0, 2, 4, and then every 4 weeks; and finally, four CD patients were treated with adalimumab (a human anti-TNF*α* monoclonal antibody) with subcutaneous injections of 160 mg at week 0, 80 mg at week 2, 40 mg at week 4, and then every 4 weeks. The choice of the specific anti-TNF*α* depended on the disease behavior and previous treatments. In particular, infliximab was chosen in UC patients and in CD patients with fistulating disease, because it was the only licensed anti-TNF*α* agent for these conditions. Certolizumab pegol and adalimumab (in two patients) were chosen for previous intolerance to infliximab. Adalimumab (in two patients) was used for luminal active CD. Moreover, all patients treated with infliximab and two of those treated with adalimumab were naïve for biological therapies, whereas all of the others had already been treated with an anti-TNF*α* with a median wash out of 52 months (range 5–84) before the assessment of Tregs in peripheral blood and the beginning of the new therapy. Other treatments ongoing at baseline were recorded, as well as the concomitant therapies during the anti-TNF*α* course. After giving informed consent, patients were studied before (T0) and 45–60 days after the beginning of anti-TNF*α* therapy (T1). Disease activity was evaluated by means of clinical activity indexes (CDAI [30] for Crohn's disease and CAI [31] for ulcerative colitis). Clinical response was defined as a decrease of CDAI of at least 70 points and of CAI of at least 4 points. We also assessed CRP levels before and after the treatment.

An ileocolonoscopy with multiple biopsies was performed before and after a median of 18 months of treatment in all patients who repeated the colonoscopy (9 CD, 1 UC) and samples were obtained from affected areas for evaluating the frequency of FOXP3^+^ Tregs in the intestinal tissues by immunohistochemistry. We also retrospectively evaluated pre- and post-treatment intestinal biopsies of 5 additional IBD patients (3 CD, 2 UC), whose peripheral blood Treg assays have not been performed. These five patients were naïve to anti-TNF*α* and were treated with infliximab as described above. Finally, we performed eight intestinal biopsies of normal mucosa in control subjects (the indication for colonoscopies was no specific abdominal symptoms; endoscopy and histology were scored as normal, and the patients were subsequently diagnosed as having irritable bowel syndrome). 

### 2.2. Laboratory Methods

#### 2.2.1. Flow Cytometry

Small aliquots of unfractionated peripheral blood were labeled with appropriate combinations of monoclonal antibodies (mAb) directed against CD4 and CD25, followed by red cell lysis, fixation, and permeabilization. Cells were then incubated for 30 minutes at 4°C with anti-FOXP3 mAb (eBioscience, USA) and analyzed on a FACS Canto flow cytometer (BD Biosciences, USA). A minimum of 20,000 events was acquired in list mode. Analyses were performed using the FACS Diva software package (BD Biosciences). Treg cells were enumerated and expressed as percentage of FOXP3^+^cells among total CD4^+^ T cells, as previously published [32]. 

#### 2.2.2. Immunohistochemistry

For identification of FOXP3^+^ lymphocytes in intestinal tissues, paraffin-embedded sections of histologically normal and CD-involved colonic biopsies were obtained before and after infliximab therapy, cut at 3-*μ*m thickness, deparaffinized, hydrated, blocked for endogenous peroxidase using 3% H_2_O_2_/H_2_O, and subsequently subjected to microwave epitope enhancement using a Dako Target retrieval solution (DakoCytomation). Incubation with the primary FOXP3 monoclonal Ab (Abcam, Cambridge, UK) was conducted for 30 min at room temperature. Detection was achieved using a standard streptavidin-biotin system (Vector Laboratories), and Ag localization was visualized with 3′-3-diaminobenzidine (Vector Laboratories). In each specimen, positive cells were expressed as the mean number of cells/high power field, calculated in a minimum of 3 high power fields (defined as a magnification ×400). 

### 2.3. Statistical Analysis

Differences between patient subgroups were assessed with two-tailed *t* test for paired (T0 versus T1) or independent (IBD versus HC) samples, as appropriate, after checking for normal distribution of data by D'Agostino-Pearson test. Frequencies were compared by Fisher's exact test. Correlations were evaluated with Spearman's rank test. *P* values < 0.05 were considered significant. 

## 3. Results

### 3.1. Peripheral Blood

The baseline characteristics of IBD patients involved in the peripheral blood study are summarized in Table 1. Seventeen men and 15 women with a median age of 33.5 years (range from 14 to 72 years) were included. The disease had been present for at least 6 months (range from 6 months to 26 years), with a median disease duration of 36 months. Six CD patients (19%) already underwent intestinal surgery. Median CDAI and a median CAI before treatment were, respectively, 223 and 9; in particular, all patients had active disease at baseline (CDAI > 150, CAI > 4). 

**Figure 1:**
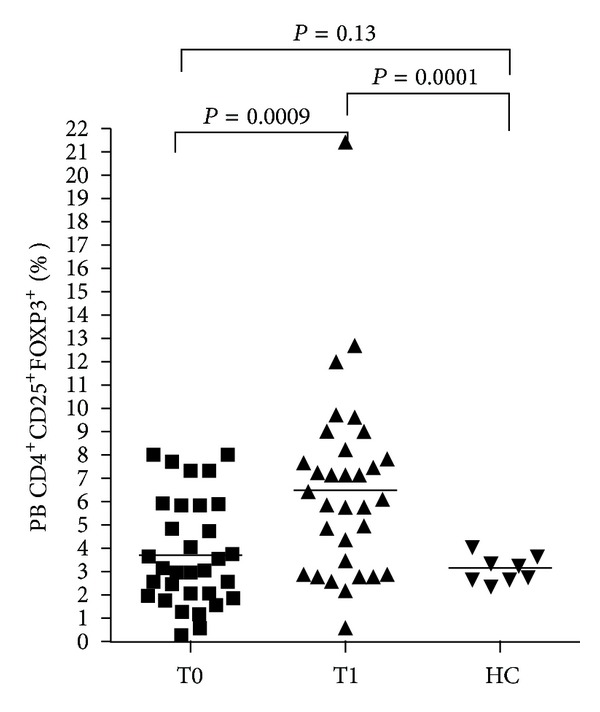
Treg frequencies in peripheral blood (PB) of all IBD patients before and after anti-TNF*α* therapy (*n* = 32); HC = healthy controls (*n* = 8); *P* values versus HC by *t* test for independent samples, T0 versus T1 by *t* test for paired samples.

In our population of IBD patients, the mean frequency of CD25^hi^FOXP3^+^ cells among total CD4^+^ T cells was significantly increased in peripheral blood (PB) after anti-TNF*α* therapy compared to T0 (Figure 1), as assessed by *t* test for paired samples (*P* = 0.0009). The mean Treg frequency of IBD patients at T0 was not different from healthy controls (HC), while at T1, it was significantly higher than HC (*P* = 0.0001 by *t* test for paired samples).

**Figure 2:**
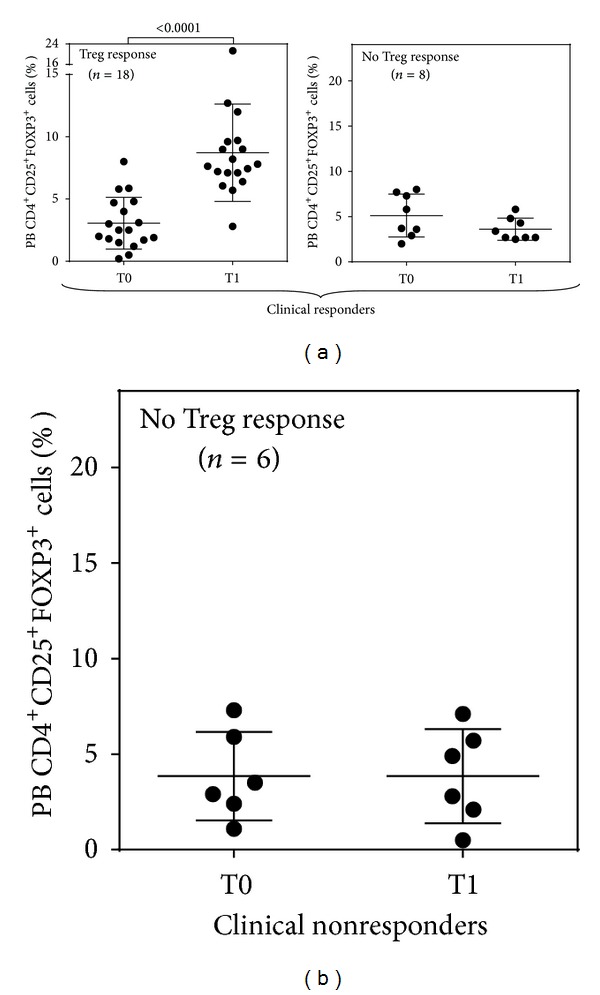
(a) “Treg responders” and “Treg nonresponders” among all IBD clinical responders; *P* value by *t* test. (b) Treg response in clinical nonresponder patients.

Twenty-six patients (21 CD, 5 UC) had a clinical response and showed a significant increase of PB Treg frequencies (*P* = 0.0006). Among the 26 clinical responders, we identified two subgroups of patients (Figure 2(a)): eighteen displaying a Treg increase and 8 without Treg response; the 6 clinical nonresponders were all Treg nonresponders (Figure 2(b)). The comparison of the number of patients displaying a Treg increase of at least 2-folds among clinical responders and nonresponders also showed a statistically significant difference (*P* = 0.0033).

**Figure 3:**
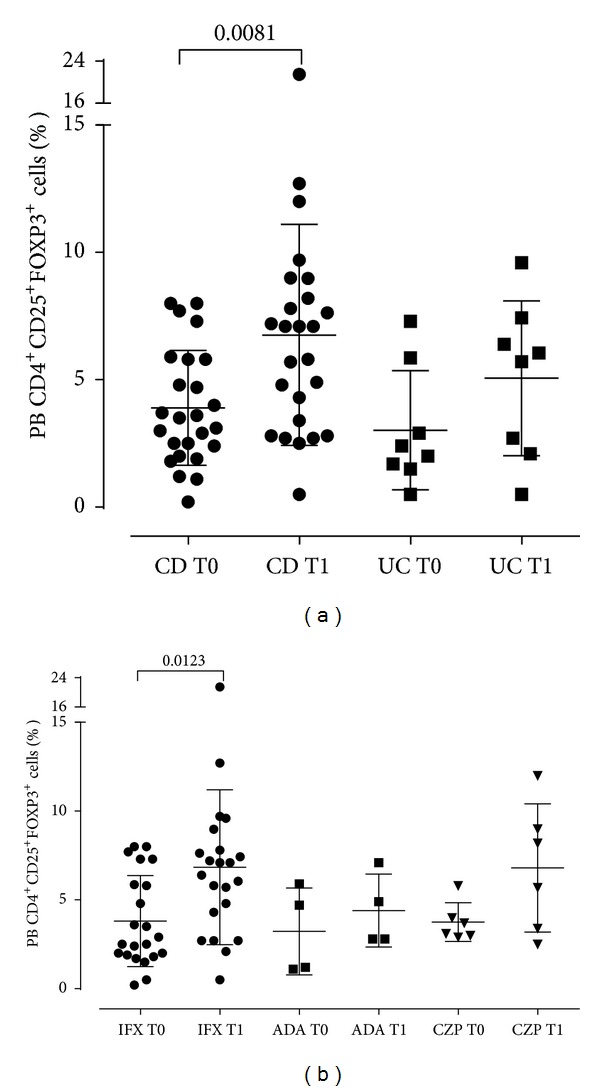
(a) Treg frequencies in Crohn's disease patients (CD, *n* = 25) and ulcerative colitis (UC, *n* = 7) before and after anti-TNF*α* therapy; *P* value by *t* test for paired samples. (b) Treg frequencies in IBD patients according to the anti-TNF*α* treatment: infliximab (IFX, *n* = 22); adalimumab (ADA, *n* = 4); and certolizumab pegol (CZP, *n* = 6); *P* values by *t* test for paired samples.

Analyzing these results in CD versus UC patients, we found in both diseases an increase of mean Treg frequencies after anti-TNF*α* treatment (Figure 3(a)), although it was statistically significant in only CD patients (*P* = 0.0081, T1 versus T0). Among clinical responders, both CD and UC patients showed an increase of Tregs at T1, which was significant for only CD patients (*P* = 0.0045).

A further analysis was done comparing the different anti-TNF*α* agents (Figure 3(b)). While a trend to an increase after treatment was evident for all treatment groups, the difference at T1 was significant only for patients treated with infliximab (*P* = 0.0123).

**Figure 4:**
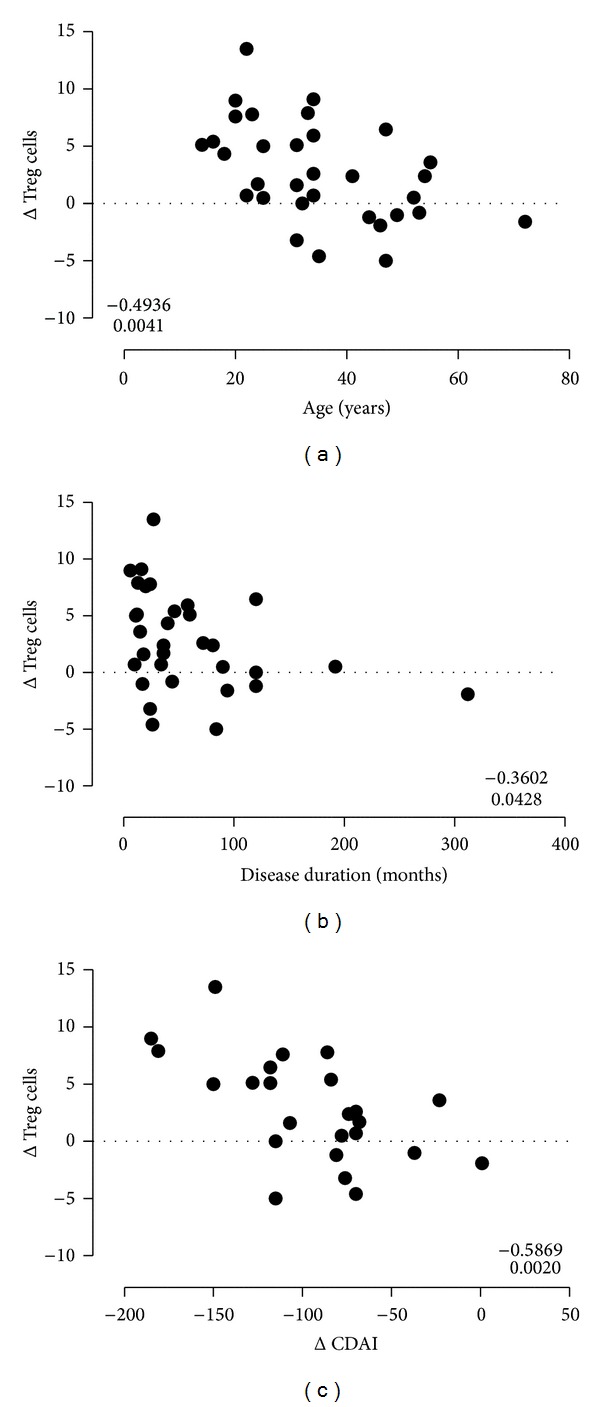
(a) Correlation between age of IBD patients and delta Treg frequencies between T1 and T0 (Spearman's rho = −0.49; *P* = 0.0041; *n* = 32). (b) Correlation between disease duration in months and delta Treg level between T1 and T0 (Spearman's rho = −0.36; *P* = 0.042; *n* = 32). (c) Correlation between delta CDAI and delta Treg level between T1 and T0 in Crohn's disease patients (Spearman's rho = −0.58; *P* = 0.002; *n* = 25).

We analysed the presence of correlations between age of patients and duration of disease (expressed in months from diagnosis) and delta Treg frequencies (defined as value at T1 − value at T0). The results (shown in Figure 4) indicated a significant negative correlation of delta Tregs and both patient's age (Figure 4(a)) and duration of disease (Figure 4(b)). While age was significantly higher in the nonresponder patients as compared to the responder ones (mean age ± standard deviation 49.8 ± 15.6 versus 31.5 ± 11 years, respectively, *P* = 0.03 by *t* test), the duration of disease was not different among the two groups (mean duration 86.3 ± 114.2 versus 52.3 ± 56.1 months, resp., *P* = 0.5 by *t* test). Taken together, these findings indicate a greater increase of Tregs in patients with disease of shorter duration. The correlation with clinical response was further analyzed in CD patients; the delta CDAI (CDAI at T1 − CDAI at T0) was negatively correlated with the delta Treg frequencies indicating greater increase of Tregs in those patients having greater decrease of CDAI after anti-TNF*α* treatment (Figure 4(c)). 

No correlation of peripheral blood Treg percentage was found with either CRP levels or previous/concomitant use of medications other than anti-TNF*α*. No severe adverse events occurred during this study.

**Figure 5:**
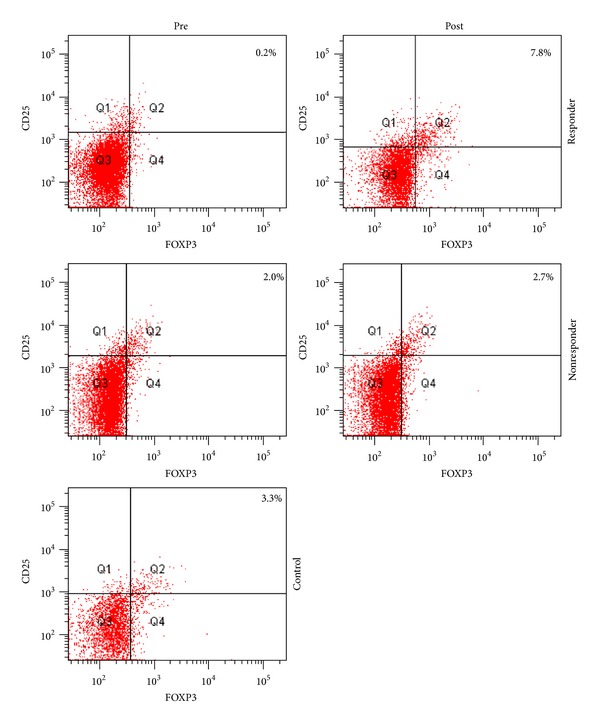
Representative dot plots of flow cytometry study. Top line left: Treg responder before anti-TNF*α*. Top line right: Treg responder after anti-TNF*α*. Middle line left: Treg nonresponder before anti-TNF*α*. Middle line right: Treg nonresponder after anti-TNF*α*. Lower line: healthy control.

Representative dot plots of flow cytometry are shown in Figure 5.

### 3.2. Lamina Propria

The characteristics of the 15 IBD patients and of the control subjects studied by immune-histochemistry are outlined in Table 2. We analyzed the expression of FOXP3 in the lamina propria (LP) of samples of intestinal mucosa obtained in 8 control subjects and in 15 patients before and after the treatment with anti-TNF*α* agents (Table 3); all of these patients showed a clinical response. Control subjects had an almost absent FOXP3^+^ cell in LP. In the biopsies of patients taken after treatment, there was a trend to an increase of mean FOXP3^+^ cell count as compared to biopsies taken at baseline in CD patients while not in the UC patients, although these differences were not statistically significant. In ten patients (8 CD and 2 UC) FOXP3^+^ cell counts were higher after treatment, while in 5 (4 CD and 1 UC), they were lower as compared to the pretreatment counts. In the ten patients of whom we gathered both PB Tregs and LP FOXP3^+^ cell counts, we did not find any correlation among these data. 

**Figure 6:**
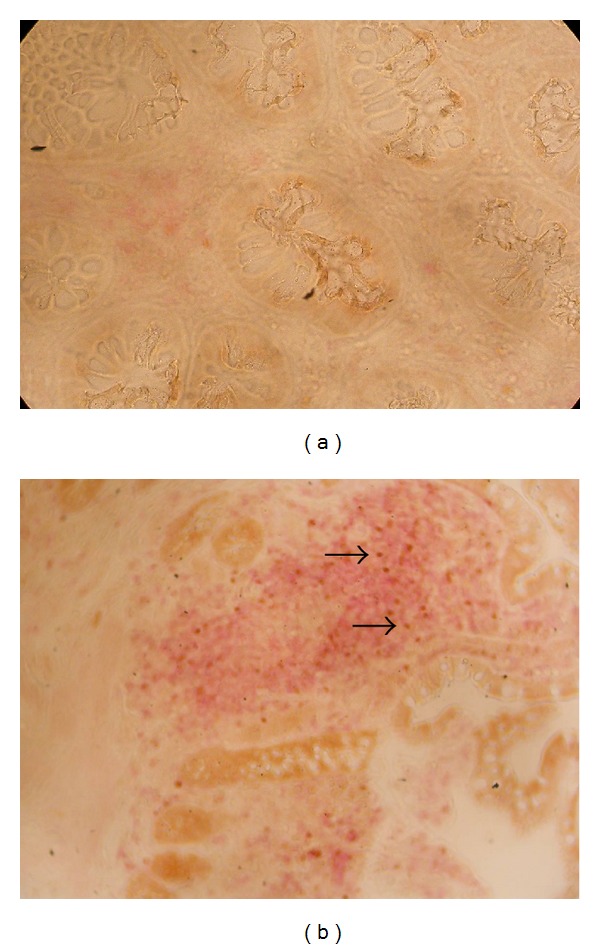
Representative immunohistochemistry slides showing FOXP3^+^ cells in colon mucosa lamina propria. (a) Before treatment with anti-TNF*α*. (b) After treatment with anti-TNF*α* (arrows show FOXP3 expressing cells).

Representative immunohistochemistry images of FOXP3^+^ lamina propria lymphocytes are shown in Figure 6.

## 4. Discussion

Upregulation of Tregs has been hypothesized as a mechanism of action of anti-TNF*α* agents. 

Our results show that IBD patients with active disease, while having no differences in circulating CD4^+^CD25^+^FOXP3^+^ Treg levels compared to healthy controls, display a significant increase of this cell type after treatment with anti-TNF*α*. Treg increase is negatively correlated with age, disease duration, and, in CD, with CDAI. Furthermore, only clinical responders to anti-TNF*α* show the increase of Tregs after therapy, while, at the tissue level, no significant changes were detected.

The first point to discuss concerns the existence of a difference in circulating Treg levels in active IBD patients compared to healthy subjects. Similar findings to ours have been described by Di Sabatino et al. who found no differences in mean Treg levels in PB between CD patients before treatment with infliximab (4.5% ± 2.7) and control subjects (3.4% ± 2.7) [22]. On the contrary, previous studies described different findings [21, 23, 26, 27]. Trying to find a possible explanation to our different results, we analyzed the baseline characteristics of our patients in comparison to those of other published studies. The median duration of disease is lower in our cohort of patients and in the group of IBD subjects analyzed by Di Sabatino et al. (3 and 4 years, resp.) [22] compared to other reports that included patients with a median duration of disease of 6–9 and 7–10 years for UC and CD, respectively [21, 23]. This suggests that a different T-cell immunoregulation may occur over time during the natural course of human IBD [15, 33] and that over time the number of FOXP3^+^ T cells may decrease. Moreover, we found that the percentage of patients who were taking immunomodulators (AZA, 6-MP) at baseline was higher in other studies [21, 23] in comparison with our cohort of patients (Table 4) and with subjects evaluated by Di Sabatino et al. who discontinued immunomodulators six months before entry into the study [22]. There are few data concerning the effects of immunosuppressors on the number and function of Tregs [34], nevertheless we may suppose that their inconsistent use could be another possible explanation for different findings in our study.

 As regards to the effects of anti-TNF*α* therapy on Tregs, our results show that there is a statistically significant increase of FOXP3^+^ T cells in PB after biological therapy compared to baseline in responder patients. Separately analyzing CD and UC patients, we found a significant increase in Treg frequency only in CD group before and after anti-TNF*α* treatment compared to UC patients. In studies conducted in patients with active rheumatoid arthritis, peripheral blood CD4^+^CD25^+^ Tregs have shown reduced FOXP3 expression and suppressive function which can be reversed by treatment with an anti-TNF*α* monoclonal antibody (infliximab) [19, 20]. These data were confirmed by a subsequent study that showed how active RA patients CD4^+^CD25^hi^ Tregs are both phenotypically and functionally altered and recover their function again after anti-TNF*α* therapy [35]. 

Some studies have been similarly performed in IBD patients. Di Sabatino et al. showed that the mean percentage of Tregs significantly increased in CD patients who responded to a 10-week treatment with infliximab (*P* < 0.0001) in comparison with the subgroup of nonresponders [22]. Boschetti et al. also documented an increase of CD4^+^CD25^+^FOXP3^+^ cells in 20 of 25 IBD patients after a single dose of anti-TNF*α*; however, they underlined that Treg frequency increased also in one nonresponder subject, and 3 of 5 IBD patients in whom peripheral Treg level did not change after anti-TNF*α* therapy were clinical responders [23]. These unclear results may be explained by considering that it is too early to analyze changes in Treg levels after only one administration of anti-TNF*α*. 

Another finding of our study is the evidence of two subgroups of clinical responder IBD patients, defined according to the “Treg response.” Eight out of 26 patients with clinical response did not have an increase of their Treg levels after the induction with anti-TNF*α*. This is similar to what has been previously described in other studies [23] and might be explained by the prevalent activation in some patients of immune pathways in the intestinal mucosa not involving FOXP3^+^ Tregs.

An important clarification about the interactions of anti-TNF*α* therapy and CD4^+^CD25^+^FOXP3^+^ Tregs came from the paper by Veltkamp et al. [25]. They showed increased apoptosis of Tregs in active IBD both in gut mucosa and in peripheral blood, with reduced levels of these cells in peripheral blood compared to controls. During anti-TNF*α* treatment, Treg apoptosis declined and peripheral Treg number was increased in correspondence with reduction of serum caspase activity of CRP and of clinical activity. On the other hand, Grundström et al. [24] demonstrated no significant impact of anti-TNF*α* treatment on Treg peripheral blood level in 34 IBD patients treated with infliximab or adalimumab, whereas analysis of T cell populations in intestinal mucosa showed an induction of effector cells. A direct comparison of these data with our study is not completely possible because some characteristics of patients, such as age and disease duration, were not specified by Grundström et al.

Our study includes the first direct comparison of the effects of different anti-TNF*α* agents on Treg levels. We found that only infliximab led to a significant increased level of peripheral CD4^+^CD25^+^FOXP3^+^ T cells, whereas Tregs did not change during treatment with adalimumab and certolizumab pegol (although the small size of these subgroups does not allow meaningful conclusions). A study on 26 CD patients treated with adalimumab found no differences in Treg frequencies in the short term, while at 26 weeks, Treg precentages increased in responders who had low Treg rates at baseline [36].

We also found an inverse correlation between age of patients and delta Tregs and between the disease duration and delta Tregs. However, the comparison of age between clinical responders and nonresponders demonstrated a statistically significant difference, leading to the possibility that the inverse correlation of Treg frequency and age may be the result of a group characteristic. On the contrary, there were no differences of disease duration between clinical responders and nonresponders. If we could consider the increase of regulatory T cells as a predictive factor of response to biological therapy, patients who receive anti-TNF*α* agents after a shorter period of time since diagnosis (“top-down” strategy [37]) may have more benefits from this therapy, confirming the possibility to change the natural course of IBD. Furthermore, this may be considered a confirmation about the hypothesis that the immunological setting changes over time in inflammatory bowel diseases [15, 33]. 

The last point regards Tregs in intestinal mucosa of IBD patients. Our data show a trend to an increase of tissue Tregs after a median of 18 months of biological therapy, although this is not statistically significant. No clear data about the normal level of tissue Tregs may be extrapolated from published studies [25–28]. Li et al. found higher tissue levels of FOXP3 in IBD patients compared to healthy controls, describing how these values decrease in patients who respond to anti-TNF*α* treatment compared to baseline [21]. On the contrary, Ricciardelli et al. recently demonstrated that in gut lamina propria of children affected by active CD, there is a reduced number of FOXP3^+^ cells compared with controls and that the frequency of Tregs and their FOXP3 expression may increase in CD children treated *in vivo* with infliximab compared with the mucosa of untreated CD children and controls [29]. 

## 5. Conclusion

Our data confirm that anti-TNF*α* treatments, in particular infliximab, may interfere with Treg levels in peripheral blood of IBD patients. Furthermore, Treg changes may be different in early versus late disease. It should be interesting to evaluate the long-term outcome of clinical responder IBD patients who do not present a significant increase in peripheral blood FOXP3^+^ Tregs; thus, a possible prognostic role may be attributed to Treg changes. Further studies are warranted to clarify the exact immunological setting of Tregs in the intestinal mucosa of IBD patients before and after anti-TNF*α* treatments, as well as the functional properties of Tregs in these patients, and in particular their suppressive ability.

## Supplementary Material

In the table 1S, peripheral blood (PB) CD4+CD25+FOXP3+ levels in all groups of patients (IBD and controls) before and after anti-TNF*α* therapy are showed.
In the figure 1S, Treg frequencies in peripheral blood (PB) of IBD clinical responders (n= 26) before and after anti-TNF*α* therapy are showed (p value by t test for paired samples).

## Figures and Tables

**Table 1:** Peripheral blood study: baseline characteristics of patients.

Variable	Results	
	*N* (%)	Median (range)
White race	32 (100)	
Male	17 (53)	
Age at entry (yr)		33.5 (14–72)
Crohn's disease	25 (78)	
Disease site		
(i) Ileum	7 (28)	
(ii) Ileocolon	17 (68)	
(iii) Colon	1 (4)	
(iv) Perianal disease	5 (18)	
Ulcerative colitis	7 (22)	
Extension		
(i) Left colitis	1 (14)	
(ii) Pancolitis	6 (86)	
Duration of disease (mo)		36 (6–312)
CDAI at T0	25 (78)	223 (155–330)
CAI at T0	7 (22)	9 (6–16)
CRP at T0 (mg/L)		5.7 (0.17–136)
Previous intestinal resection in CD patients	6 (19)	
Medications at baseline		
(i) Corticosteroids ± 5-ASA	11 (34)	
(ii) Azathioprine ± 5-ASA	9 (28)	
(iii) 5-ASA/Sulfasalamine	5 (16)	
(iv) None	7 (22)	
Medications during anti-TNF*α* therapy		
(i) Corticosteroids	1 (3)	
(ii) AZA	21 (66)	
(iii) None	10 (31)	
Anti-TNF*α*		
(i) Infliximab	22 (69)	
(ii) Adalimumab	4 (12)	
(iii) Certolizumab pegol	6 (19)	

CDAI: Crohn's disease activity index; CAI: colitis activity index; CRP: C-reactive protein; 5-ASA: 5-aminosalycilic acid; TNF: tumor necrosis factor; AZA: azathioprine.

**Table 2:** Immunohistochemical study: baseline characteristics of IBD patients and controls.

Variable	Results	
	*N* (%)	Median (range)
IBD Patients	15 (100)	
(i) Crohn's disease	12 (80)	
(ii) Ulcerative colitis	3 (20)	
White race	15 (100)	
Male	11 (73)	
Age		33 (16–64)
Disease duration (months)		59 (13–264)
Medications at baseline		
(i) Azathioprine	3 (20)	
(ii) Steroids	4 (27)	
(iii) Mesalamine	2 (13)	
(iv) Steroids + immunosuppressors	2 (13)	
(v) None	4 (27)	
Anti-TNF*α*		
(i) Infliximab	5 (33)	
(ii) Infliximab + azathioprine	7 (47)	
(iii) Certolizumab pegol + azathioprine	3 (20)	
Histological sample location		
(i) Colon	7 (47)	
(ii) Ileum	8 (53)	
Control patients (IBS)	8 (100)	
Male	2 (25)	
Age		38.5 (15–66)
Histological sample location		
(i) Colon	0	
(ii) Ileum	8 (100)	

IBD: inflammatory bowel disease; TNF: tumor necrosis factor; IBS: irritable bowel syndrome.

**Table 3:** Lamina propria (LP) FOXP3^+^ cells*.

Subjects (*n*)	Mean LP FOXP3^+^ cell count before anti-TNF*α* (T0)	SD	Mean LP FOXP3^+^ cell count after anti-TNF*α* (T1)	SD	*P* values T0 versus T1^∧^	*P* values T0 versus controls^#^	*P* values T1 versus controls^#^
All IBD (15)	4.03	3.92	5.69	7.04	0.26	0.0015	0.0077
Crohn's disease (12)	4.64	4.02	6.97	7.36	0.19	0.0022	0.0076
Ulcerative colitis (3)	1.59	2.75	0.59	0.52	0.64	0.43	0.002
Controls (8)	0.038	0.1					

*Count/high power field.

^∧^By *t* test for paired samples.

^#^By *t* test for independent samples.

IBD: inflammatory bowel disease; TNF: tumor necrosis factor; SD: standard deviation.

**Table 4:** Concomitant medications at baseline: comparison among studies on peripheral blood Treg frequencies in inflammatory bowel diseases.

Authors	Steroids	IM	Steroids + IM
Guidi et al.	34%	28%	None
Di Sabatino et al. [22]	NA	Discontinued 6 months before the study	NA
Li et al. [21]	20%	42.5%	27.5%
Boschetti et al. [23]	56%	63% (CD), 33% (UC)	NA

IM: immunomodulators; CD: Crohn's disease; UC: ulcerative colitis; NA: not applicable.
