# Supplementary material for: FOXP3+ T Regulatory Cell Modifications in Inflammatory Bowel Disease Patients Treated with Anti-TNFα Agents
Source: Biomed Res Int. 2013 Aug 26;2013:286368. doi: 10.1155/2013/286368 (PMC3766994; doi:10.1155/2013/286368)
Supplement: Supplementary file 1 — In the table 1S, peripheral blood (PB) CD4+CD25+FOXP3+ levels in all groups of patients (IBD and controls) before and after anti-TNFα therapy are showed. In the figure 1S, Treg frequencies in peripheral blood (PB) of IBD clinical responders (n= 26) before and after anti-TNFα therapy are showed (p value by t test for paired samples). [file 286368.f1.pdf]

Table IS. Peripheral blood (PB) $CD4^{+}CD25^{+}FOXP3^{+}$ levels (TNF: Tumor Necrosis Factor, IBD: Inflammatory Bowel Disease).

Subjects (n)	Mean PB Tregs before anti-TNFα (T0)	SD^	Mean PB Tregs after anti-TNFα (T1)	SD^	P values T0 vs T1*	P values T0 vs controls #	P values T1 vs controls #
All IBD (32)	3.05	2.29	6.52	4	0.0009	0.13	0.0001
Crohn's Disease (25)	3.86	2.25	6.75	4.33	0.0081	0.093	0.0003
Ulcerative Colitis (7)	3.10	2.5	5.71	2.6	0.13	0.94	0.0326
Clinical responders (26)	3.69	2.33	7.14	4.07	0.0006	0.2	0.0001
CD responders (21)	4.02	2.31	7.31	4.39	0.0045	0.08	0.0003
UC responders (5)	2.31	2.06	6.43	2.5	0.064	0.48	0.04
Clinical non responders (6)	3.85	2.31	3.85	2.47	0.99	0.43	0.46
Controls (8)	3.03	0.58					

ˆStandard Deviation

*by t test for paired samples

by t test for independent samples

LEGEND FOR SUPPLEMENTARY FIGURE:

Figure 1S. Treg frequencies in peripheral blood (PB) of IBD clinical responders (n= 26) before and after anti-TNFα therapy; p value by t test for paired samples.

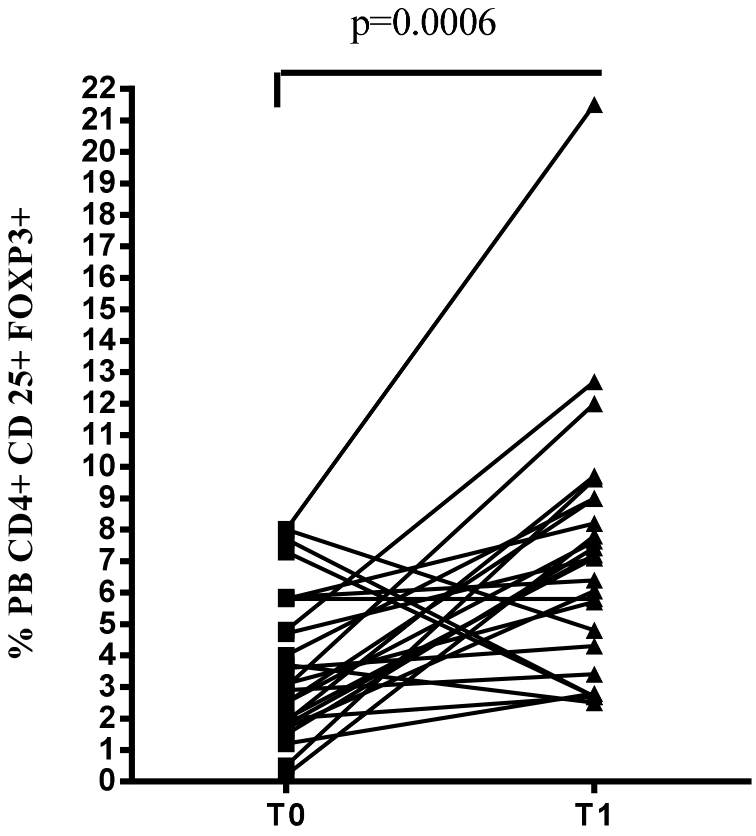
p=0.0006
22
21
20
19
18
17
16
15
14
13
12
11
10
9
8
7
6
5
4
3
2
1
0
% PB CD4+ CD 25+ FOXP3+
T0
T1

Figure 1S
